# A low-cost, wireless, 4-channel EEG measurement system used in virtual reality environments

**DOI:** 10.1016/j.ohx.2024.e00507

**Published:** 2024-01-24

**Authors:** Zhiyuan Yu, Shengwen Guo

**Affiliations:** a Department of Biomedical Engineering, School of Materials, South China University of Technology, Guangdong Province, China; b Department of Intelligent Science and Engineering, School of Automation, South China University of Technology, Guangdong Province, China

**Keywords:** Electroencephalogram, Virtual reality, Wearable device, Biopotential measurement

## Abstract

The combination of Virtual Reality (VR) technology and Electroencephalography (EEG) measurements has shown tremendous potential in the fields of psychology and neuroscience research. However, the majority of EEG measurement devices currently available are expensive, bulky, uncomfortable to wear, and difficult to integrate with VR headsets. These limitations have hindered the development of related research fields. This study describes a low-cost (60.07 USD), small-sized, wireless, high-precision, low-power consumption 4-channel EEG measurement system (NeuroVista) for frontal area EEG measurements, which can be used with a VR headset, enabling EEG measurements in VR environments. The system has an input-referred noise of less than 0.9480 μVrms, a common mode rejection ratio of over 96 dB, a measurement resolution of less than 0.1 μV, a bandwidth of 0.5 ∼ 45 Hz, and works at a sampling rate of 250 Hz. It also supports metal dry electrodes and includes a built-in analog bandpass filter, right-leg drive circuit, and built-in digital lowpass filter and notch filter, which can reduce noise during measurement. Researchers can reconstruct the electrode system to measure regions of interest according to their needs.

## Graphical abstract

**Figure:**
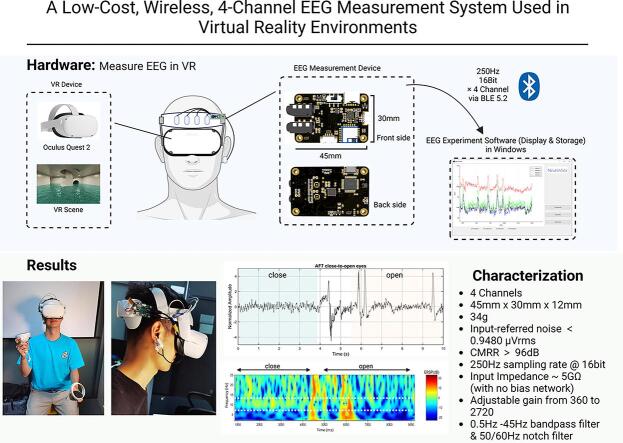

## Introduction

Specifications table.

**Table:** 

Hardware name	*NeuroVista*
Subject area	Engineering and materials science Medical Neuroscience Biological sciences
Hardware type	Biomedical engineering
Closest commercial analog	OpenBCI Galion
Open source license	CC BY 4.0
Cost of hardware	60.07 USD
Source file repository	Mendeley Data: http://doi.org/10.17632/4gfb6g46kb.2

## Hardware in context

The electroencephalogram (EEG) is a widely used technique for estimating changes in neurophysiological activity associated with external stimuli and/or the execution of specific tasks [1]. Mechanistically, when resident neurons in the brain are excited, they produce a biopotential that can be recorded by the electrodes of an EEG measurement device [2], [3]. These potentials reflect the sum of post-synaptic activities in the cerebral cortex caused by synchronous cortical neurons [4], which are associated with various functions, including motor, sensory, and cognitive functions [5].

Recently, Virtual Reality (VR) technology has promoted the development of a new neurophysiological research paradigm. VR is a medium that employs specialized hardware and sensory feedback to create a virtual experience surrounding the user, making the perception and sensation of the virtual world comparable to the physical world [6]. VR can effectively block external interference, provide highly controllable visual inputs, and due to its immersive nature, elicit stronger emotional arousal in subjects than traditional stimuli [7], [8]. Consequently, VR is widely used in psychology and cognitive science research, with an increasing number of researchers attempting to shift neurophysiological testing into the VR environment [9], [10], [11].

However, the majority of current EEG measurement devices are expensive, bulky, uncomfortable to wear [12]. Moreover, integrating them with VR equipment is challenging. These limitations hinder the advancement of research in the relevant field. Typically, devices require extremely high input impedance (>10MΩ), high common-mode rejection ratio (>80dB), and minimal input-referred noise (<6μVpp) [13]. Consequently, they necessitate high-performance analog front-end and high-precision analog-to-digital converters (ADCs), which contribute to the high cost. Furthermore, the currently widely-used EEG analog front-end chip packages have relatively large dimensions (e.g., TI's ADS129X series [14], TQFP64, 12.2 mm × 12.2 mm), making it difficult to integrate them into VR headsets and further limiting the commercialization of research findings.

Therefore, exploring and applying advanced analog front-end is necessary. In this study, we utilized a cost-effective bio-potential measurement chip - the KS1092, to develop a low-cost, wireless EEG measurement system (NeuroVista), comprising both the measurement hardware and accompanying software, for recording frontal 4-channel EEG signals.

The reason for choosing the frontal area is as follows: the frontal lobe cortex, particularly the anterior part of the frontal lobe (prefrontal cortex), plays a central role in cognitive control and executive functions. These functions include planning, decision-making, and emotion regulation [15]. For instance, in one study, EEG from the frontal lobe region was used for the screening of mild cognitive impairment and mild dementia [16]. Improving the asymmetry between left and right frontal brain alpha activity through neurofeedback is considered an effective method for treating depressive disorder [17], [18]. Attention monitoring systems often use EEG from a single frontal channel [19]. For research areas like cognitive function screening and mental disorder diagnosis, a 4-channel system can provide sufficient information. Table 1 lists EEG studies conducted in the frontal lobes with a small number of electrodes (≤6). The naming of electrode positions follows the international 10–20 system [20] (Table 1).

**Table 1:** EEG studies conducted in the frontal lobe.

Ref.	Research aspect	Electrode positions	Number of electrodes
Peeters *et al*[17], 2013	Impact of neurofeedback on mood	F3, F4, C3, C4, P3, P4	6
Hu *et al*[21], 2015	Mental stress assessment	Fp1, Fp2, Fpz	3
Zheng *et al*[22], 2016	Anxiety assessment	Fpz	1
Carvalo *et al*[23], 2020	Epileptic encephalopathy	C3, C4	2
Frankel *et al*[24], 2021	Remote seizure monitoring	F7, F8, T5, T6	4
Liang *et al*[19], 2022	Attention monitoring system	Fpz	1
Lee *et al*[16], 2022	Screening for cognitive impairment and dementia	AF3, AF4, AF7, AF8, Fp1, Fp2	6

The NeuroVista system works with Oculus Quest 2, assisting researchers to conduct psychological and neuroscience experiments within virtual reality environments. The entire system is open-source, allowing researchers to customize it according to their specific requirements.

**Fig. 1:**
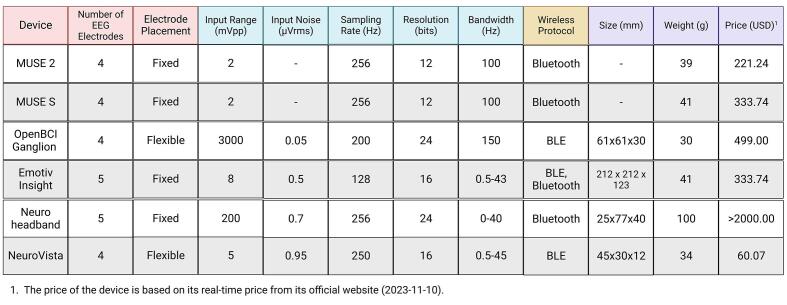
Specifications of EEG devices.

For researchers in the field of neuroscience, there is a variety of portable EEG measurement devices available on the market. NeuroVista stands out by offering a novel open-source hardware design approach that integrates EEG measurement with VR devices. Fig. 1 compares several parameters of existing EEG measurement devices [25], including the Number of EEG Electrodes, Electrode Placement (Fixed or Flexible), Input Range (mVpp), Input-Referred Noise (μVrms), Sampling Rate (Hz), Resolution (bits), Bandwidth (Hz), Wireless Protocol, Size (mm), Weight (g), and Price (USD). In similar devices, NeuroVista has the smallest size and the lowest price.

## Hardware description

**Fig. 2:**
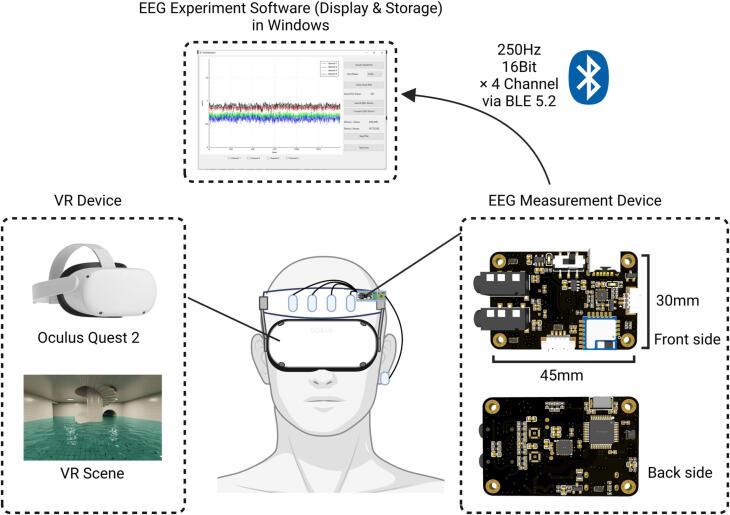
Schematic diagram of the proposed EEG measurement system (NeuroVista).

The EEG measurement system (NeuroVista) consists of three components: the EEG measurement device, VR device, and EEG experiment software (Fig. 2). The EEG measurement device is designed to capture biopotential signals from the body, perform filtering, amplifying, and digitizing the signals into digital data. This data is then packaged and transmitted to the host computer via Bluetooth Low Energy (BLE). The VR device creates an interactive virtual reality environment for the user. The EEG experiment software is specifically developed to receive, display and store the data transmitted from the EEG measurement device. It provides researchers with a user-friendly interface to visualize and analyze the EEG signals in real-time or offline.

The EEG measurement device of NeuroVista is capable of measuring EEG signals from four frontal channels, with a reference electrode placed at the earlobe. Additionally, it employs a right-leg driven electrode to reduce common-mode noise in the human body, which is also placed at the earlobe. The device is compact, measuring 45 mm x 30 mm x 12 mm, and its weighs of 34 g, allow for easy attachment to VR headset. It is powered by a 3.7 V lithium battery with an integrated power management module, and it can be conveniently recharged using a USB type-c 5 V power adapter. With a power-efficient design, the device has minimal power consumption and supports continuous usage for over 2 h with 250mAh Li-ion battery.

**Fig. 3:**
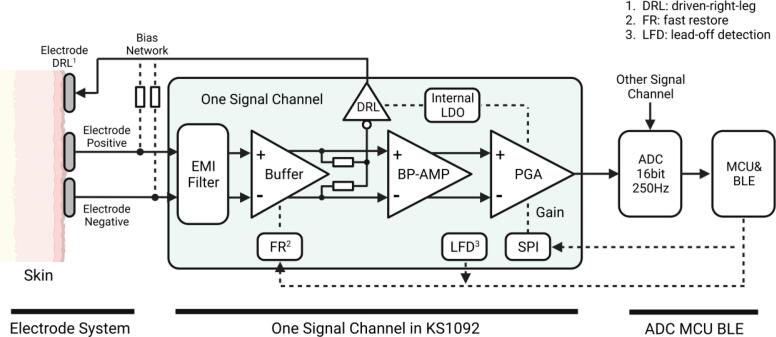
Signal path of the EEG measurement device.

The transmission path of the single-channel EEG signal is illustrated in Fig. 3. It consists of three components: the electrode system, the signal path in KS1092, and the signal conversion and transmission unit comprising an ADC (AD7682, Analog Devices, Inc.), a Microcontroller Unit (MCU, STM32F103C8T6, STMicroelectronics N.V.), and a Bluetooth Low Energy module (BLE106, Jinan USR IOT Technology Ltd. China).

Firstly, the electrode system plays a crucial role in directly contacting the skin to collect the differential potential of the human body's surface. Additionally, the electrode system is responsible for loading the inverted common-mode noise output from the driven-right-leg circuit onto the human body, reducing common common-mode noise interference.

Subsequently, the collected EEG signals are acquired, filtered, and amplified using KS1092 (Shenzhen Kingsense Electronics Co., Ltd. China). KS1092 is an integrated analog front end for EEG or other weak biopotential measurements, with two differential signal channels. It includes programmable gain amplifiers (PGAs), a 0.05 ∼ 200 Hz band-pass amplifier (BP-AMP), a driven-right-leg circuit (DRL), a digital serial peripheral interface (SPI), and an internal high precision low dropout regulator (LDO). It has two-stage PGAs, with a total amplification factor adjustable ranging from 360 to 2720 times. The KS1092 amplifies the biopotential into signals with amplitudes ranging from 0 V to 1.8 V. Two KS1092s are employed in the NeuroVista system to form 4-channels.

Then, the signals output by the KS1092 are collected and converted by AD7682. The AD7682 is used as the ADC, which is a 16-bit, 4-channel successive approximation register analog-to-digital converter (SAR ADC). The AD7682 features an internal reference power supply that can be set to 2.5 V. This eliminates the need for an external high-precision reference voltage source.

NeuroVista incorporates a battery management system (BMS) that is built around the BMS chip BQ24073, a product of Texas Instruments (TI). Users can recharge the device using a USB adapter through type-c port. However, it is important to note that charging introduces significant noise into the signal chain. Therefore, it is advised to avoid conducting EEG measurements while the device is being recharged.

The model of the MCU is STM32F103C8T6, manufactured by STMicroelectronics. It has an ARM 32-bit Cortex-M3 core with a frequency of 72 MHz, 64 Kbytes of Flash memory, and 20 Kbytes of SRAM. Additionally, it is equipped with 37 general-purpose input/output (GPIO) pins. The functions of the MCU are as follows:

1. Configuring the parameters of KS1092 and AD7682.

2. Reading the conversion results of AD7682 at fixed time intervals.

3. Conducting digital signal processing on EEG data, which includes low-pass filtering and notch filtering.

4. Encapsulating EEG data and sending it to the BLE module via UART, which then transmits the data to the host computer.

**Fig. 4:**
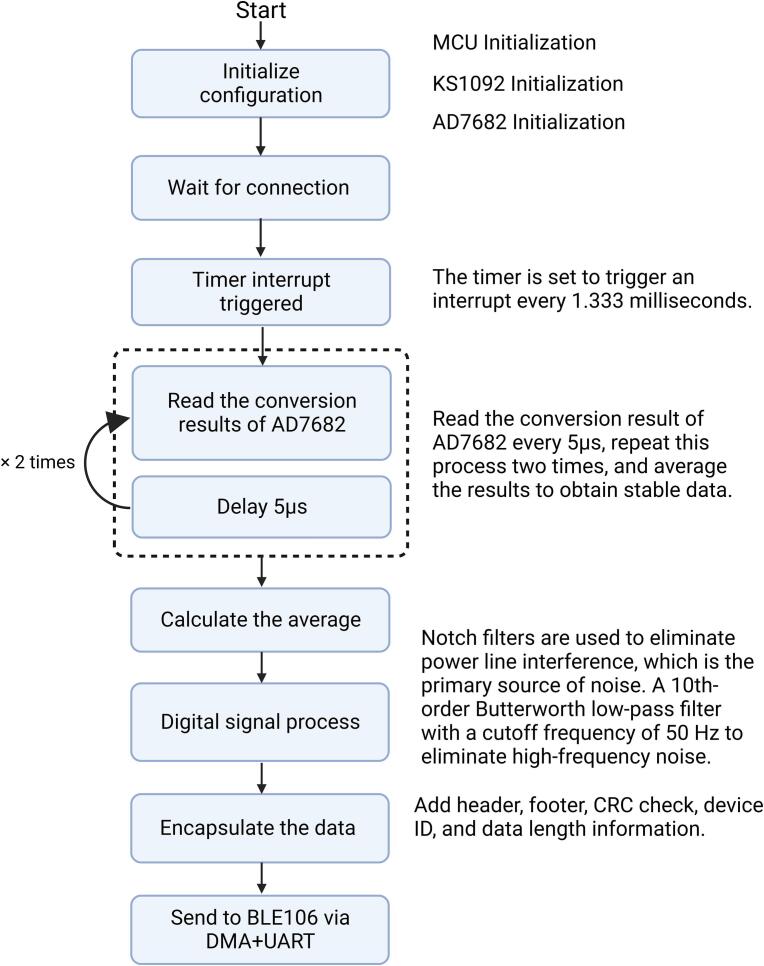
The flowchart of the program on the MCU.

The flowchart of the program on the MCU is shown in Fig. 4.

**Fig. 5:**
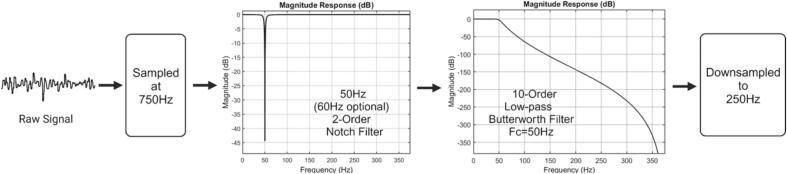
The flowchart of the digital signal processing on the MCU.

The EEG signals are initially sampled at a rate of 750 Hz, followed by processing through a 2nd-order single notch filter at 50 Hz/60 Hz with bandwidth of 8 Hz to eliminate power line interference present in the signal. Thereafter, the signals are subjected to a 10th-order Butterworth low-pass filter with a cutoff frequency of 50 Hz to eliminate high-frequency noise. Finally, the signals are downsampled to 250 Hz. This process involved initially sampling at a higher rate followed by downsampling, primarily because a higher sampling rate is more effective in combating aliasing effects [26]. The use of filters at a higher sampling rate allows for more precise signal processing. However, due to the processing speed limitations of the MCU and the constraints of the BLE module, transmitting data at a rate of 250 Hz is nearly reaching the system's limit. Therefore, the signal, after digital signal processing, is downsampled to 250 Hz (Fig. 5).

NeuroVista is capable of providing high-precision EEG data. Its input-referred noise is less than 0.9480μVrms, with a common-mode rejection ratio greater than 96dB, input impedance (with bias network) approximately 20MΩ, and input impedance (without bias network) approximately 5GΩ. The sampling rate is 250 Hz, with a sampling resolution of less than 0.1 μV.

In the application case presented in this article, NeuroVista supported Oculus Quest 2 VR headset. It is recognized as one of the most popular VR headsets on the market [27], with a wide range of community resources, but it does not include eye-tracking technology. NeuroVista provides researchers with the flexibility to interchange the VR headset and electrode system based on their specific requirements. To facilitate communication between the EEG measurement devices and the host computer, a dedicated software application has been developed. This software runs on the Windows platform and enables the display and storage of EEG signals. Furthermore, methods for accessing and subscribing to BLE are provided, implying that any device compatible with BLE can access and retrieve EEG data uploaded by NeuroVista through UUID (Universally Unique Identifier).

NeuroVista offers several advantages that can assist researchers in achieving their research objectives:

1.High-precision EEG data: NeuroVista provides EEG measurements with low input noise, high common-mode rejection ratio, and suitable input impedance, ensuring researchers to obtain accurate and reliable EEG signals, facilitating precise analysis of brain activity.

2.Compatibility with VR device: The NeuroVista system is compatible with VR headset, thus allows researchers to integrate virtual reality environments into their experiments. This opens up opportunities for conducting cognitive experiments, neurofeedback training, and immersive simulations, enhancing the realism and versatility of their research.

3.Portable and wireless: The Bluetooth Low Energy technology of NeuroVista's EEG measurement device enables wireless communication with the host computer, making it convenient for researchers to conduct experiments without being restricted by wired connections.

4.Low cost: The average per-channel cost of NeuroVista is lower than that of other existing EEG measurement devices [25].

## Design files

Electronics files, software files, and testing records can be found in the following public repository: Mendeley Data: http://doi.org/10.17632/4gfb6g46kb.2. This work is under the CC BY 4.0 open source license (Table 2).

**Table 2:** Design file summary.

Design file name	File type	Open source license	Location of the file
SCH NeuroVista Mainboard.json	schematic file	CC BY 4.0	Schematic and PCB files folder
PCB NeuroVista Mainboard.json	PCB file	CC BY 4.0	Schematic and PCB files folder
NeuroVista Mainboard PCB Gerber.zip	Gerber file	CC BY 4.0	Schematic and PCB files folder
PickAndPlace Mainboard PCB NeuroVista.csv	Pick&Place File	CC BY 4.0	Schematic and PCB files folder
NeuroVista Shield Board PCB Gerber	Gerber file	CC BY 4.0	Schematic and PCB files folder
PCB NeuroVista Shield Board.json	PCB file	CC BY 4.0	Schematic and PCB files folder
NeuroVista Init STM32.rar	STM32CubeIDE Project	CC BY 4.0	STM32 software folder
NeuroVista main STM32.rar	STM32CubeIDE Project	CC BY 4.0	STM32 software folder
NeuroVista QT Software Project.rar	QT Project	CC BY 4.0	Disply&Storage Software folder
NeuroVista Software Release.rar	QT Project	CC BY 4.0	Disply&Storage Software folder
NeuroVista Software Developer's Guide.pdf	Pdf file	CC BY 4.0	Disply&Storage Software folder
BOM NeuroVista.csv	csv file	CC BY 4.0	BOM folder

Design files description:

SCH NeuroVista Mainboard: The schematic file of main board, open it using EasyEDA.

PCB NeuroVista Mainboard: The PCB file of main board, open it using EasyEDA.

NeuroVista Init STM32: Configuration file burned when the first-time use. Open it using STM32CubeIDE 1.6.1.

NeuroVista main STM32: Complete embedded project files. Open it using STM32CubeIDE 1.6.1.

NeuroVista QT Software Project: Display and storage software, QT project on the Windows platform, open it using QT Creator. QT version 6.4.3, compiled with MSVC2019_64.

NeuroVista Software Release: The release version of the display and storage software can be used directly after extracting.

BOM NeuroVista: Complete BOM file, includes details of the motherboard's components along with purchase links for each component. The main supplier for electronic components is LCSC Electronics.

## Bill of materials

Only partial information of important components is listed, and the complete BOM file can be found in the public repository: Mendeley Data: http://doi.org/10.17632/4gfb6g46kb.2 (Table 3).

**Table 3:** Bill of materials summary.

Component	Number	Cost per unit (USD)	Total cost (USD)	Source of materials
Main Board^1^	1	22.99	22.99	
Silver-Woven Fabric Electrode	4	1.37	5.48	https://item.taobao.com/item.htm?spm=a1z09.2.0.0.44c02e8dvqfw3q&id=677903683078&_u=333r9ooifb7c
Ear Electrode Clamp	1	8.23	8.23	https://item.taobao.com/item.htm?spm=a1z09.2.0.0.44c02e8dvqfw3q&id=561114449471&_u=333r9ooi3b0f
ECG Lead Wire	4	2.06	8.24	https://item.taobao.com/item.htm?spm=a1z09.2.0.0.44c02e8dod1DNZ&id=654892666581&_u=333r9ooi9e4b
3.5 mm 4P audio Plug	2	0.27	0.54	https://item.taobao.com/item.htm?spm=a1z09.2.0.0.44c02e8dvqfw3q&id=672376474562&_u=333r9ooic0b4
VR Holder^2^	1	12.75	12.75	https://item.taobao.com/item.htm?spm=a1z09.2.0.0.44c02e8d6goQc4&id=660487198909&_u=333r9ooi9789
M2 Bolt (M2*4 + 3)	8	0.011	0.088	https://detail.tmall.com/item.htm?_u=333r9ooid60b&id=723347838083&spm=a1z09.2.0.0.44c02e8d6goQc4
M2 Nut	4	0.044	0.176	https://detail.tmall.com/item.htm?_u=333r9ooi915e&id=612966794798&spm=a1z09.2.0.0.44c02e8d6goQc4&skuId=4315124529170
Lithium-ion Battery (250mAh 3.7 V)	1	1.75	1.75	https://item.taobao.com/item.htm?spm=a1z09.2.0.0.44c02e8dvqfw3q&id=528235852444&_u=333r9ooi8732

1. The complete BOM of the mainboard can be found in the attached BOM file

2. In addition to the aforementioned components, a VR device is also required. The VR holder in Table 3 supports Oculus Quest 2, and if other VR devices are used, the VR holder can be replaced accordingly.

3. The cost does not include PCB manufacturing and SMT expenses

## Build instructions

### Component Introduction

**Fig. 6:**
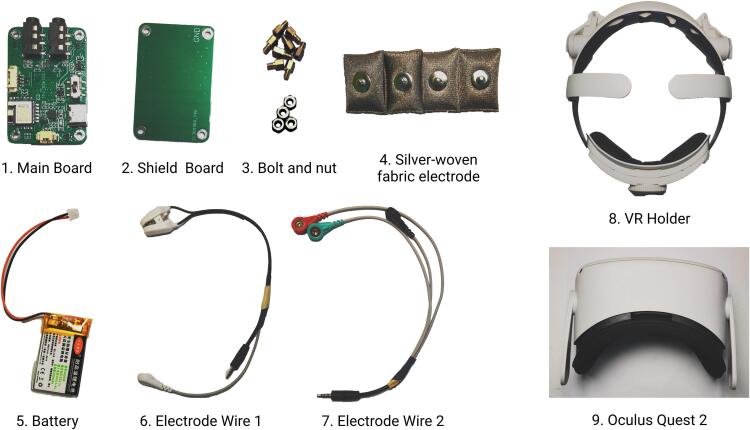
Components of NeuroVista.

Fig. 6 presents all components of NeuroVista. Prepare these components before assembly.

**Fig. 7:**
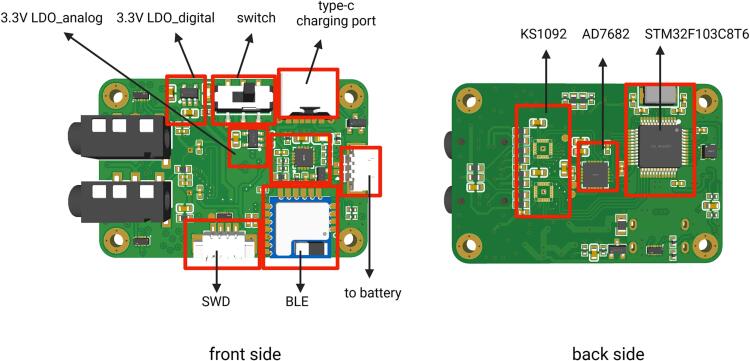
Layout of main board.

**1. Main Board**: The main board is equipped with key components for EEG measurement, including modules such as the analog frontend, ADC, MCU, and BLE. The digital and power conversion components are located on the front side, while the precision analog components are positioned on the back side. The layout of modules is shown in Fig. 7. Produce it according to the design file [SCH NeuroVista Mainboard.json] and [PCB NeuroVista Mainboard.json], open them using EasyEDA [28].

The manufacture requirements for the mainboard PCB are as follows: a 4-layer structure, FR4 board type, outer copper thickness of 1 oz, inner copper thickness of 0.5 oz, minimum linewidth ≤ 0.2 mm, minimum line gap ≤ 0.2 mm, and minimum via diameter ≤ 0.3 mm.

The file [NeuroVista Mainboard PCB Gerber.zip] contains detailed PCB information for the mainboard, which should be sent to a PCB manufacturer for the production of the mainboard PCB. All components for NeuroVista are mounted using Surface Mounted Technology (SMT), and it is advisable to engage an SMT contractor for component assembly. The file [PickAndPlace Mainboard PCB NeuroVista.csv] provides essential details on the positioning and orientation of the components, which, along with the PCB information from [NeuroVista Mainboard PCB gerber.zip], guides the SMT contractor in the assembly process.

**2. Shield Board**: A shield board is a grounded copper foil, used to isolate external electromagnetic radiation, while also serving as the base for the mainboard. Manufacture the PCB for the shield board according to [NeuroVista ShieldBoard PCB Gerber.zip]. The manufacturing process requirements for the shield board are identical to those of the mainboard. Since the shield board does not contain any components, SMT is not required.

**3. Bolts and Nuts**: The mainboard and the shield board are fastened together through standoffs and nuts. All screw hole sizes of NeuroVista are M2

**4. Silver-Woven Fabric Electrode**: The surface of the electrode is a layer of silver-woven fabric material, with polyester fiber fillings inside. This structure endows it with excellent elasticity, making it very comfortable for users to wear. On the back of the electrode, there is a buckle that can be attached to the ECG electrode connector.

**5. Battery**: A rechargeable lithium battery with a capacity of 250mAh and a voltage of 3.7V. The interface is a 2P interface with a pitch of 1.25mm.

**6. Electrode Wire Set 1**: This set of electrode wires includes a reference electrode, a DRL electrode, and an electrode for Channel-4. The reference and DRL electrodes are mounted on a clamp that is attached to the earlobe, and are made of silver chloride. The electrode wire for Channel-4 is made using medical ECG electrode wire, with a groove at the top that allows for the attachment of a silver woven cloth electrode. Electrode Wire Set 1 is connected to the main board via a 3.5 mm 4P audio plug.

**Fig. 8:**
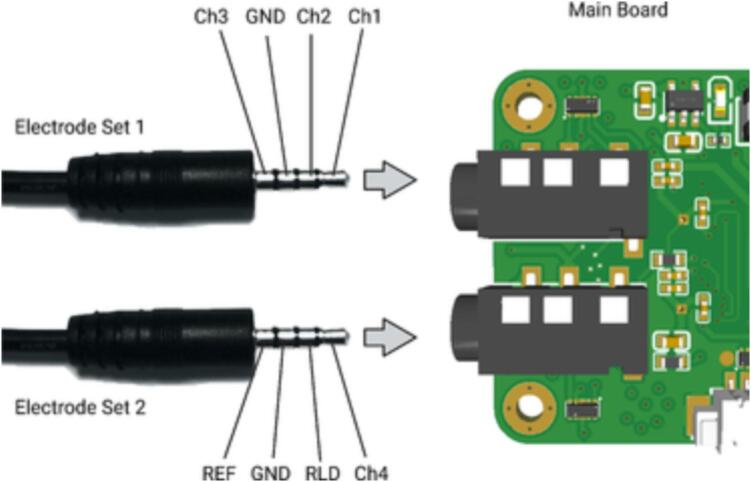
The relationship between the audio plug and the electrodes.

**7. Electrode Wire Set 2**: This set of electrode wires includes electrodes for Channel-1, Channel-2, and Channel-3. The electrode wires are made from medical ECG electrode wire, and the top end has a groove that can accommodate a silver fabric electrode. Electrode Wire Set 2 connects to the main board via a 3.5 mm 4P audio plug. The relationship between the audio plug and the electrodes is illustrated in Fig. 8.

**8. VR Holder**: A plastic VR holder, used to hold VR headset on head. There are various types of VR holders available in the market, offering different features and designs. It is recommended to choose a VR holder that best suits the needs and is convenient for purchase.

**9. Oculus Quest 2**: A VR headset. Researchers can change the VR headset according to their experimental platform

## Assembly process

**Fig. 9:**
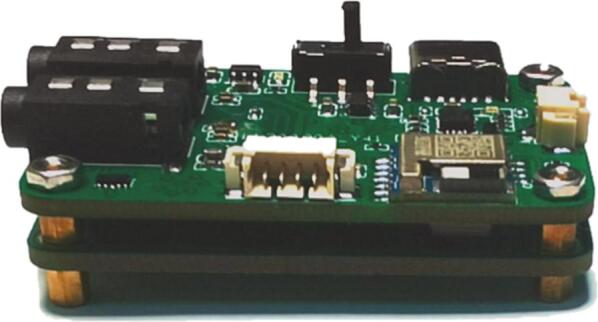
The assembly form of the main board and shield board.

**Fig. 10:**
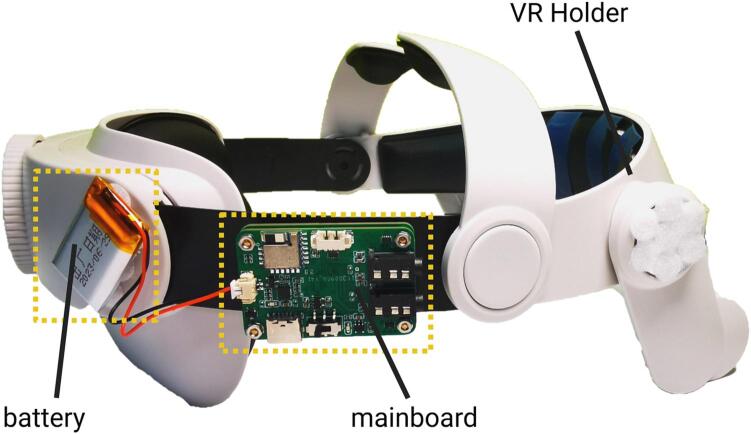
Assembly schematic of the mainboard, battery, and VR holder.

**Fig. 11:**
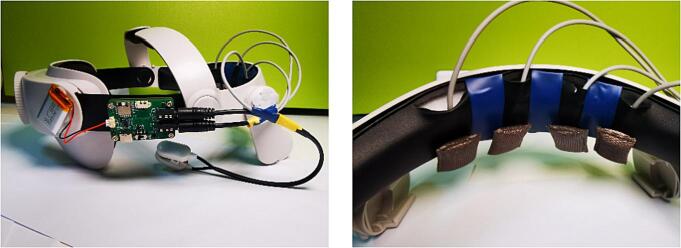
The electrode placement for NeuroVista.

**Fig. 12:**
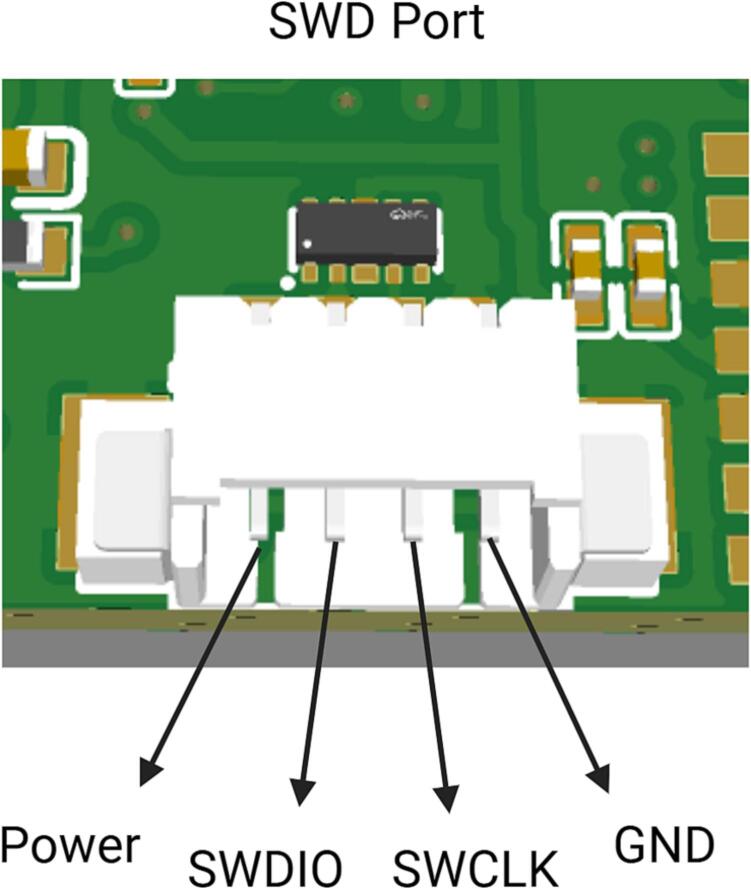
SWD port description.

1. Combine the mainboard and the shield board together using bolts and nuts (Fig. 9).

2. Adhere the mainboard and the battery to the VR holder (Fig. 10).

3. Arrange the electrode wires on the VR holder and install the silver fabric electrodes (Fig. 11).

4. For the initial use, burn the configuration program [NeuroVista Init STM32] for the BLE106 module into the MCU (STM32F103C8T6). The purpose of this program is to set the UART baud rate of the BLE106 module to 500000bps. Burn and run the program, then restart the device. The program on the MCU is developed based on STM32CubeIDE 1.6.1. We recommend the following burning method: download and install STM32CubeIDE 1.6.1 or a higher version, open the project file [NeuroVista main STM32] in the STM32CubeIDE environment, and burn using JLink or STLink in the way of SWD. The interface of SWD is 4P 1.25 mm (Fig. 12).

5. Burn the main program. Following the method in step 4, burn the main program [NeuroVista main STM32] into the MCU.

6. The assembly process is complete.

## How to modify the electrode system according to research requirements

In biopotential measurement tasks, the quality of the recorded signals strongly depends on the electrodes used.

When electrodes conduct biopotential signals, they come into direct contact with an electrolyte solution, such as conductive gel, human sweat, or tissue fluid, thereby forming a metal-electrolyte interface [29]. As a result, a potential difference known as the half-cell potential or Nernst potential arises due to the charge distribution difference between the metal and the solution. This phenomenon is called electrode polarization [30]. When using two electrodes to measure the potential between two points in a biological system, artifacts may be introduced due to differences in the polarization level of the two electrodes [29]. These artifacts can be reduced through electrode material selection or denoising algorithms. An excellent biomedical electrode material is the silver-silver chloride electrode. Silver chloride exhibits extremely low solubility in water, allowing the electrode and its surrounding electrolyte to maintain a relatively stable microenvironment. Consequently, the polarization potential in this setup is minimal and remains constant.

**Fig. 13:**
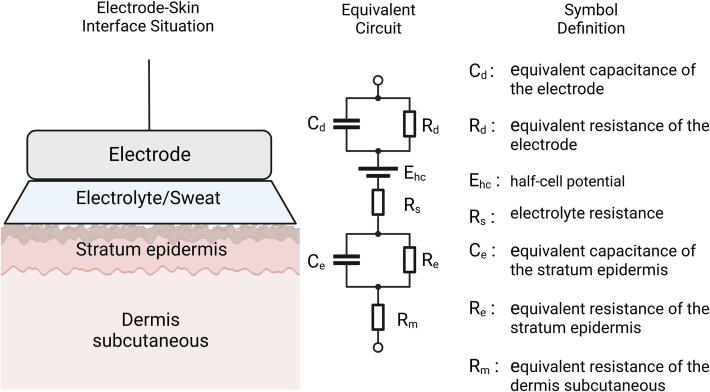
Equivalent circuit of the electrode–skin interface.

Impedance is another crucial property to consider when selecting electrodes. Kaczmarek and Webster proposed a well-known electrode–skin interface equivalent circuit model [31], which quantitatively evaluates the impedance of the electrodes (Fig. 13). According to this model, electrodes with different materials and sizes exhibit significant differences in impedance and half-cell potential. In this study, dry electrodes are used, eliminating the need for conductive gel and providing a more comfortable user experience. However, the impedance of dry electrodes can reach several tens of kΩ at low frequencies. Therefore, it is crucial to ensure the preamplifier used in the system has high enough input impedance to mitigate the decrease in common-mode rejection ratio resulting from differences in electrode impedance.

**Fig. 14:**
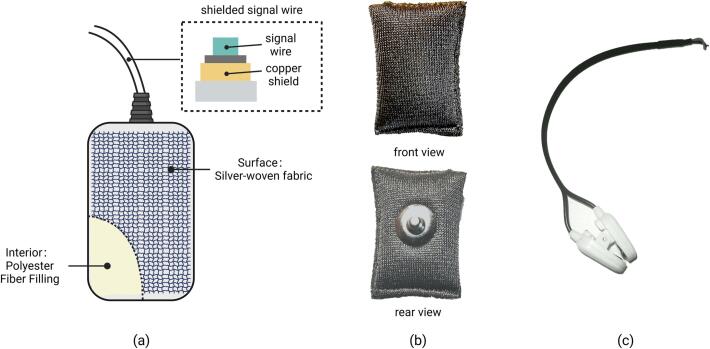
Electrodes used in NeuroVista: (a) Schematic of the silver fabric electrode; (b) Physical image of the silver fabric electrode; (c) Physical image of the ear electrode clamp.

The electrode system utilized in NeuroVista incorporates two different types of electrodes to fulfill specific functions, the elastic silver fabric electrodes used to measure EEG signals on the forehead (Fig. 14.b), and the silver-silver chloride electrodes clipped to the earlobe, serving as reference and driven-right-leg electrodes (Fig. 14.c). The surface of the silver fabric electrode is covered with a layer of silver fabric material filled with polyester fibers (Fig. 14.a). This structure provides good elasticity and ensures comfortable use for the wearer. The impedance of all electrodes is kept below 10kΩ at 10Hz. The electrode wires are of a dual-layer structure, with an outer layer of grounded copper foil shielding and an inner layer of signal wire. This design effectively reduces electromagnetic noise on the wires. Researchers have the flexibility to reconstruct the electrode system according to their specific needs. However, it is strongly recommended to incorporate a shielding layer to the conductors. This additional shielding layer will further enhance the system's ability to isolate external electromagnetic noise and maintain signal integrity [32]. The shielding layer is grounded at one end, while the other end remains unconnected.

## How to configure KS1092 according to research requirements

The EEG signals are acquired, filtered, and amplified using the KS1092.

A pull-up resistor of 20MΩ is connected to the Driven-Right-Leg (DRL) port, forming a voltage bias network. In fact, in the vast majority of application scenarios, there is no need for a dedicated voltage bias network, resulting in an input impedance of approximately 5GΩ. If the contact impedance of the electrode system is excessively high, the pull-up resistor can be removed to achieve a higher input impedance.

The KS1092 adopts band-pass amplifier. Due to ultra-low cutoff frequency of the high-pass filtering characteristic, signal may require several seconds to settle. This settling time can cause an undesirable delay for the user when the electrodes are first connected. To address this issue, the fast restore pin (PIN8: FR) of the KS1092 can be controlled by an external signal. By setting PIN8: FR to a high state, the fast recovery mode is activated. Consequently, a low-resistance loop path between the input and output of the BP-AMP is established, leading to a faster settling time.

**Fig. 15:**
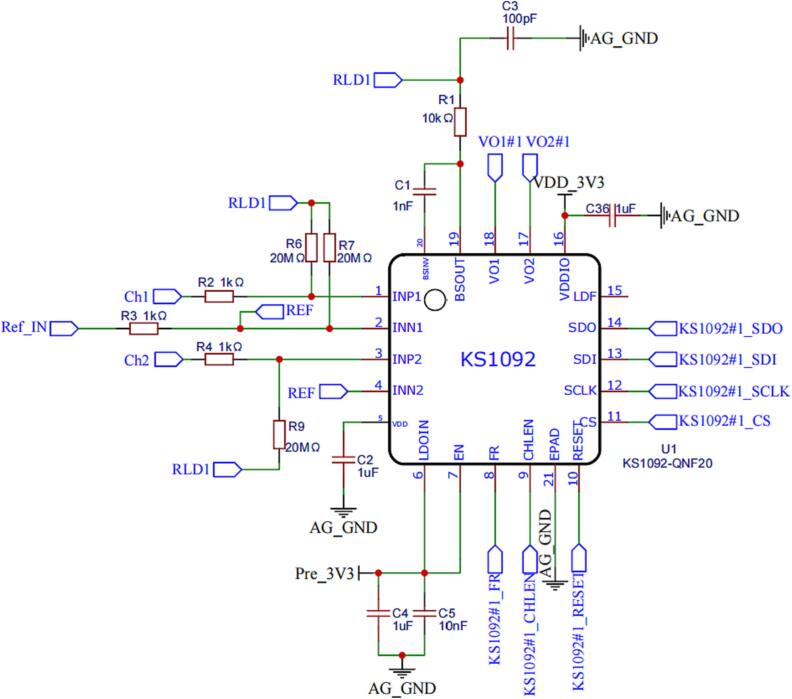
Peripherals circuit of KS1092.

DRL is used as a means to counter the common-mode interference in a biopotential measurement system caused by power line and other source. The principle of DRL involves perceiving the common-mode interference from the positive and negative input ends, then inversely amplifying it, and loading it onto the human body. This creates a negative feedback loop between the device and the human body, effectively restricting the common-mode interference [33]. The KS1092 includes a built-in DRL, with PIN19: BSOUT serving as the output terminal of the DRL. When using the DRL, a capacitor should be connected between PIN19: BSOUT and PIN20: BSINV. In practical applications, a 1-nF capacitor (C1) is commonly used. **For safety considerations, a resistor (R1) is connected between the DRL electrode and DRL output (PIN19: BSOUT) to limit the current flowing into the human body (**Fig. 15**). The value of this resistor ranges between 10kΩ and 100kΩ.**

## Operation instructions

**Fig. 16:**
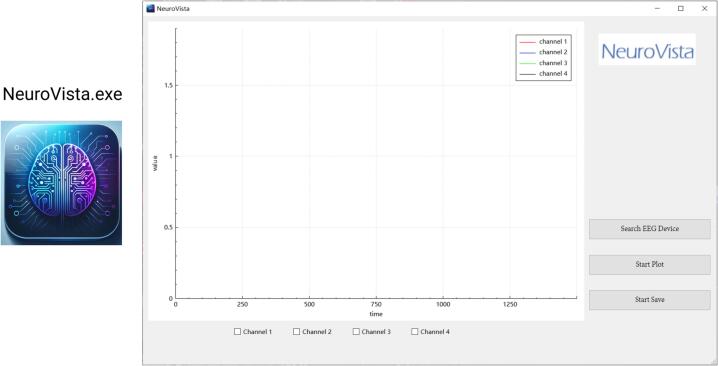
The icon and interface of NeuroVista’s software.

**1.** Before utilizing NeuroVista, extract the contents of [NeuroVista QT Software Release.zip]. Following this, initiate the software by clicking on “NeuroVista.exe” (Fig. 16). It is important to note that NeuroVista's software is currently compatible only with the Windows platform. Should you wish to use it on a different platform, or if you are interested in further development, guidance can be found in the provided [NeuroVista Software Developer's Guide.pdf]. The subsequent sections will detail the usage workflow of NeuroVista.

**2.** Prior to utilizing NeuroVista's software, ensure that the computer is equipped with support for Bluetooth Low Energy (BLE). BLE was integrated into the Bluetooth standard and has been supported since the release of Bluetooth version 4.0. Devices with Bluetooth 4.0 or higher are guaranteed to have BLE capabilities.

**Fig. 17:**
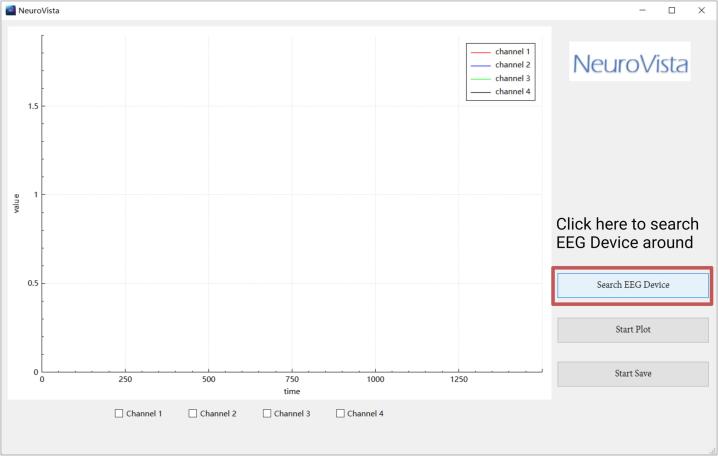
Click on 'Search EEG Device' to search for nearby NeuroVista devices.

**Fig. 18:**
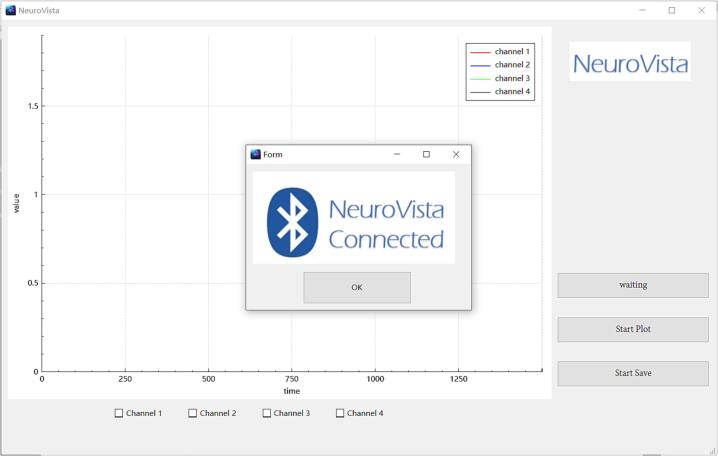
After the software successfully connects to a device, a notification window will pop up.

**3.** Power on NeuroVista, and wait for a 5-second initialization period. Then, click on 'Search EEG Device' to search for nearby NeuroVista devices (Fig. 17). When a device is detected, the software will automatically connect to it and a notification window will pop up (Fig. 18).

**Fig. 19:**
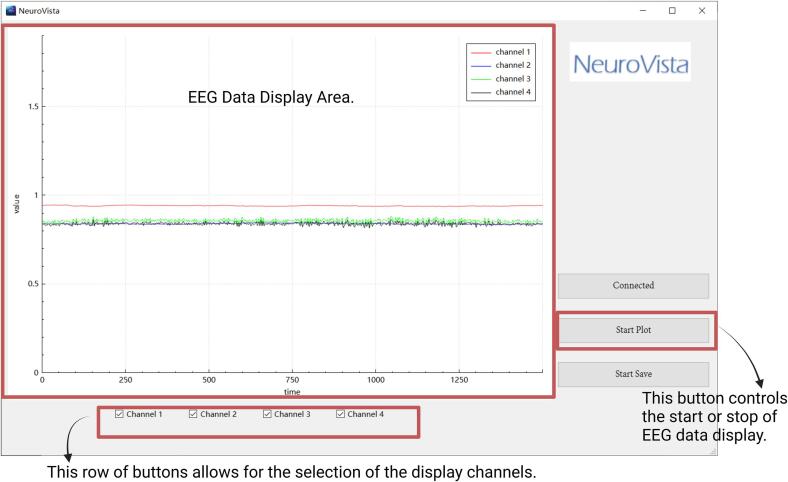
Operating methods of the EEG data display.

Refer to Fig. 19 for the relevant operating methods of the EEG data display.

**Fig. 20:**
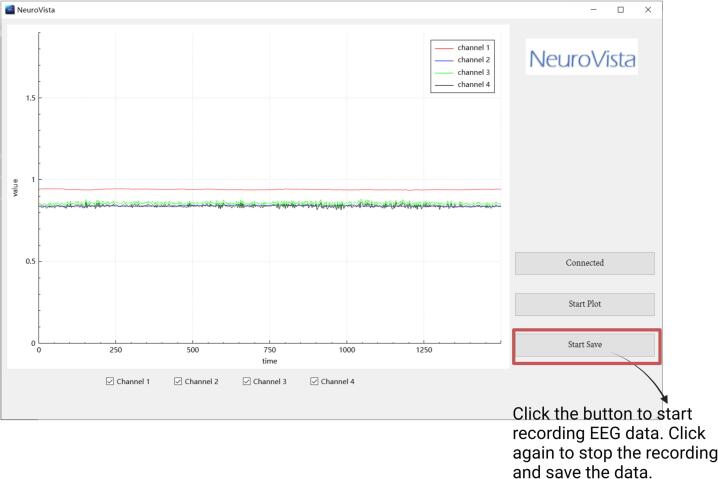
Instructions for the Storage of EEG Data.

**Fig. 21:**
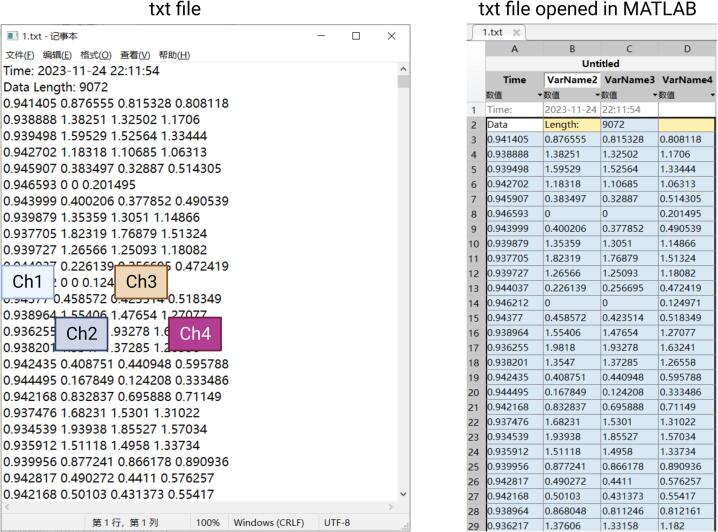
The txt file generated by NeuroVista.

**5.** Click the button to start recording EEG data; click again to end the recording and save it (Fig. 20). The EEG data will be saved as a txt file, which includes the date and time of measurement, the number of sampling points, and the EEG data itself (Fig. 21). This txt file can be read by software such as MATLAB, Excel, Origin, etc.

**Fig. 22:**
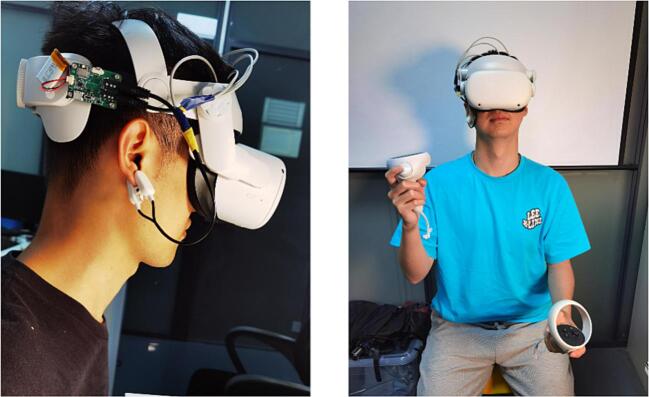
Subjects wearing NeuroVista.

Equip the research subject with NeuroVista (Fig. 22). During this process, pay close attention to the contact situation of the electrodes.

**Fig. 23:**
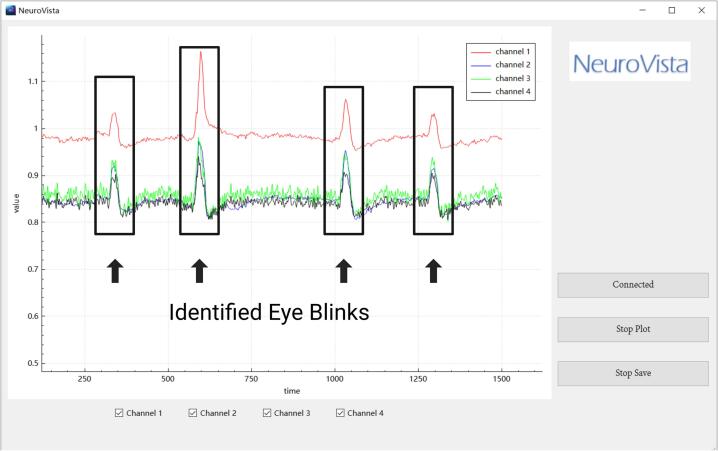
Identified eye blinks in EEG waveform.

**7.** This introduces a simple, rapid, yet effective method for assessing the quality of electrode contact. After the subject has donned the device, they are asked to blink a few times. If the electrodes are positioned close to the eyes, eye movement artifacts will appear on the EEG display (Fig. 23). Generally speaking, the greater the amplitude of the eye movement artifact, the better the quality of electrode contact.

## Validation and characterization

To demonstrate the system's performance, NeuroVista was tested under conventional laboratory conditions, at an ambient temperature of 25 °C, in an indoor environment without any signal shielding measures, powered by a 3.7 V lithium battery, the frequency of the power line notch filter has been set to 50 Hz, data rate = 250 SPS, gain is 720 (unless otherwise noted).

Test items and parameter requirements refer to the industry standard IEC 80601–2-26:2019 for commercial electroencephalographs.

### Input-referred noise

**Fig. 24:**
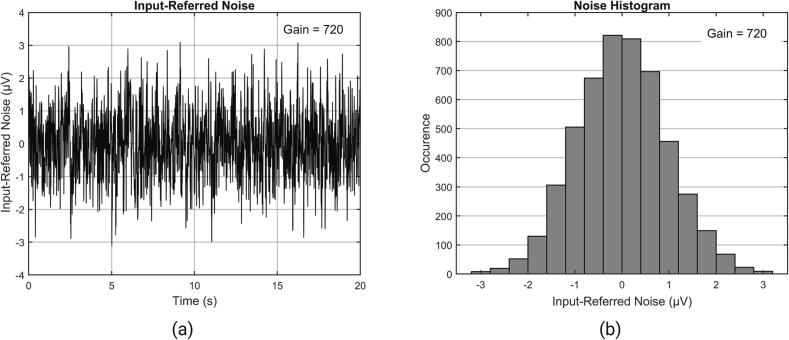
(a) Input-referred noise waveform (b) Input-referred noise amplitude distribution.

Due to the extremely small amplitude of EEG signals, which are often measured in microvolts (µV), it is essential for the system to have low levels of noise. According to common design benchmarks, the input noise for each channel should not exceed 6 μVpp. To verify the system's input noise, the input channel of the EEG measurement device is connected with the reference end (Ref), the peak to peak (Vpp) and root mean square (Vrms) of the noise voltage is recorded when they are short-circuited. During this short-circuit condition, the system employs a gain of 720 times. Fig. 24 illustrates the signal of one channel in this particular setup. By analyzing a noise signal of 20 s in length, it was determined that the system's input-referred noise is Vrms<0.9480μV (Vmax=3.0978μV, Vmin=-3.1095μV, Vpp=6.2073μV, $\mu =3.6570\times 10^{-5}\;\mu V$, σ=0.9476μV). The Vrms calculated using the following formula:

$$Vrms=\sqrt{\frac{1}{N}\sum_{i=1}^{N}{V(i)}^{2}}$$

where: V(i) is the voltage value at the i-th sample point. N is the total number of sample points.

### Frequency response

According to IEC 80601–2-26:2019, when measuring sinusoidal signals, the EEG measurement device should meet the frequency response or bandwidth requirements of 0.5 Hz to 50 Hz. The NeuroVista supports a bandwidth of 0.5 Hz to 45 Hz. Ideally, the output at frequencies of 0.5 Hz and 45 Hz should be within the range of 71 %∼110 % of the output obtained from a 5 Hz sinusoidal input signal.

A set of test sinusoidal signals, with frequencies ranging from 0.05 Hz to 100 Hz and each having a peak-to-peak amplitude of 1 mV, is generated by a signal generator and applied between the input channel and the reference end of NeuroVista. The signal generator used in this experiment is the UTG932, manufactured by UNI-Trend Technology (China) Co., Ltd. This instrument boasts an amplitude output range of 1mVpp to 10Vpp (≤10 MHz), amplitude flatness of ± 0.1 dB (≤100 kHz), and a frequency output range from 1μHz to 30 MHz.

**Fig. 25:**
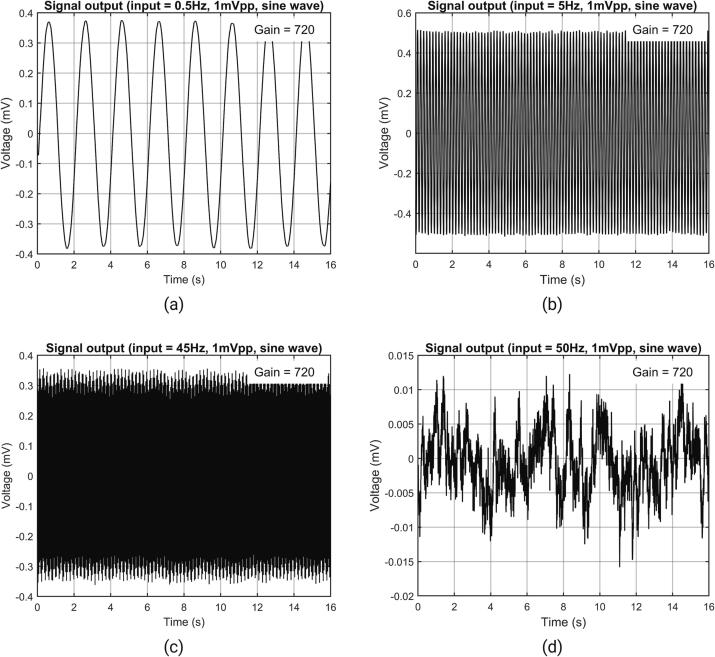
(a) Waveform of 0.5 Hz sinusoidal signal (b) waveform of 5 Hz sinusoidal signal (c) waveform of 45 Hz sinusoidal signal (d) waveform of 50 Hz sinusoidal signal.

**Fig. 26:**
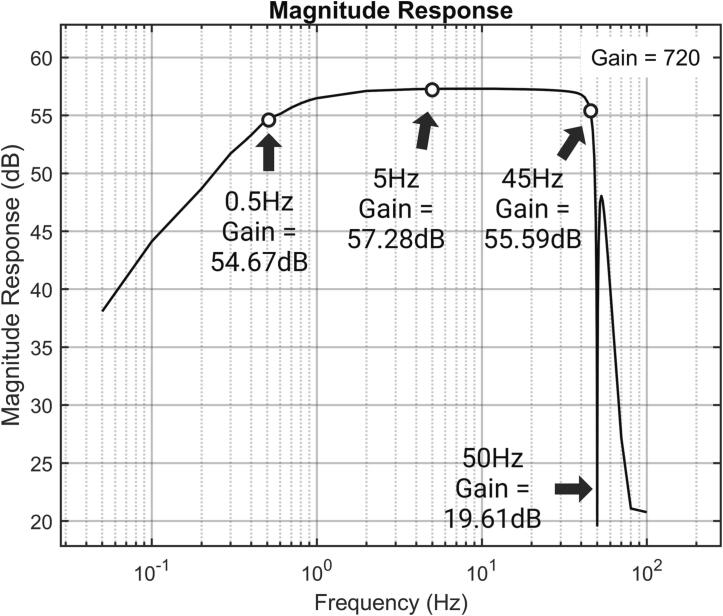
Frequency response curve.

During testing, it was observed that the amplitude of the 0.5 Hz signal was 74.1450 % Vrms of the amplitude of the 5 Hz signal, while the amplitude of the 45 Hz signal was 82.3529 % Vrms of amplitude of the 5 Hz signal (Fig. 25). For ease of presentation, the voltage values of the output signal have been adjusted to the referred input voltage according to the gain. The complete frequency response curve is shown in Fig. 26.

### Common mode rejection ratio

The Common Mode Rejection Ratio (CMRR) reflects a system's ability to suppress common mode signals. CMRR is typically expressed in decibels (dB) and can be calculated using the following formula:

$$CMRR\left(dB\right)=20\times \log_{10}\left(\frac{V_{rms,diff}}{V_{rms,cm}}\right)$$

Where:

$V_{rms,diff}$ is the Vrms value of the differential mode signal.

$V_{rms,cm}$ is the Vrms value of the common mode signal.

In the case of an EEG device, it is typically required to have a CMRR greater than 80 dB. To measure the system's CMRR, the input channel was connected to the reference end, and then the common mode signals were introduced through a resistance–capacitance network composed of a 51kΩ resistor and a 47nF capacitor. According to the IEC 80601–2-26:2019, at this point, the power line notch filter was turned off.

**Fig. 27:**
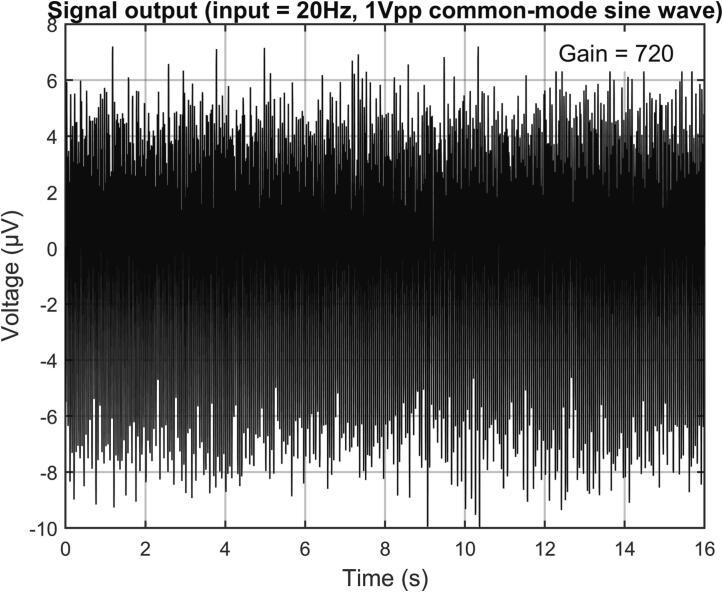
The output waveform when the input signal is a 20 Hz 1Vpp sinusoidal common-mode signal.

**Fig. 28:**
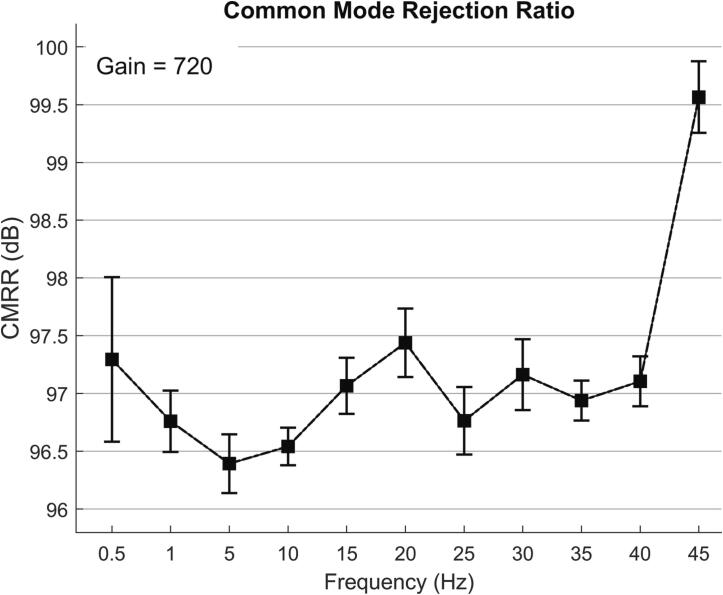
The relationship between CMRR and frequency.

The common mode signals were sinusoidal waves with frequencies of 0.5 Hz, 1 Hz, 5 Hz, 10 Hz, 15 Hz, 20 Hz, 25 Hz, 30 Hz, 35 Hz, 40 Hz, and 45 Hz, respectively, and the peak-to-peak value was 1 V. Fig. 27 shows the output waveform when the input signal was 20 Hz. For ease of presentation, the voltage values of the output signal have been adjusted to the referred input voltage according to the gain. At each frequency, the output signal is measured eight times over 16 s, and their mean and error are calculated. The relationship between CMRR and frequency is shown in Fig. 28. The measured CMRR of the system is found to be greater than 96 dB.

### Open-Eye and Closed-Eye experiment

To demonstrate that the EEG measurement device collects brainwave signals rather than noise, an open-eye and closed-eye experiment was conducted. In this experiment, the focus was on recording the alpha rhythm of EEG signals which typically falls within the frequency range of 8–14 Hz. The alpha rhythm is prominent in the occipital and parietal lobes and can be detected at any position on the head [34]. It is known to appear when individuals close their eyes and enter a relaxed state [35]. A control experiment was designed to record brainwave forms using the EEG measurement device in both closed-eye and open-eye states. The power spectrum analysis was performed on the collected data to explore and verify the practical performance of the EEG device. This experimental setup allows for the evaluation of the EEG measurement device's ability to capture and differentiate brainwave signals from noise, specifically focusing on the alpha and theta rhythm in the open-eye and closed-eye states.

A total of three subjects participated in the experiment, all of whom were male, with ages 23, 24, and 26 years, respectively. They had normal vision and hearing, were right-handed, and had no history of psychiatric treatment. The experiment was conducted in a quiet, enclosed environment. For each subject, EEG data were collected in both open and closed eye states, each comprising six segments of 20 s.

Every subject has signed an informed consent before the experiment. The informed consent includes an introduction to the experiment, potential experimental risks, the researcher's obligations and rights, the subject's obligations and rights, compensation for participating in the experiment, and procedures for handling research-related injuries. Subjects have the right to withdraw from the experiment at any stage without any obligation.

**Fig. 29:**
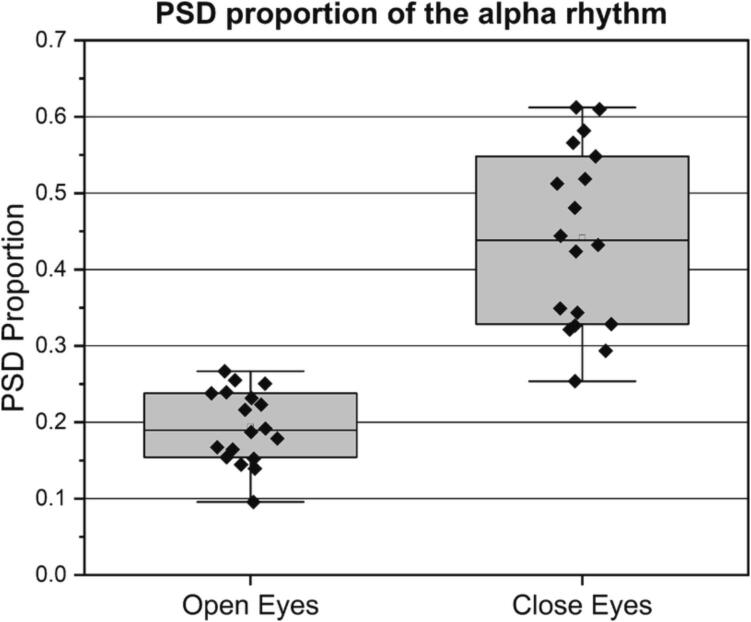
PSD proportion of the alpha rhythm.

The power spectral density (PSD) proportion is used to quantify the intensity of the alpha rhythm. Its calculation method is: the PSD integrated over the alpha rhythm frequency range (8–14 Hz) divided by the PSD integrated over the entire frequency range (0.5–45 Hz). There are 18 cases of EEG data in the eyes-open state and 18 cases of EEG data in the eyes-closed state. The PSD proportion charts generated from these data are shown in Fig. 29.

In the *t*-test analysis, a significant increase in the intensity of the alpha rhythm was observed when comparing eyes-closed state (μ=0.4414, σ=0.1137) to eyes-open state (μ=0.1942, σ=0.0467),t(34)=8.53, p<0.0001, consistent with the results of neuroscience research, demonstrating that NeuroVista captured effective brain activity.

**Fig. 30:**
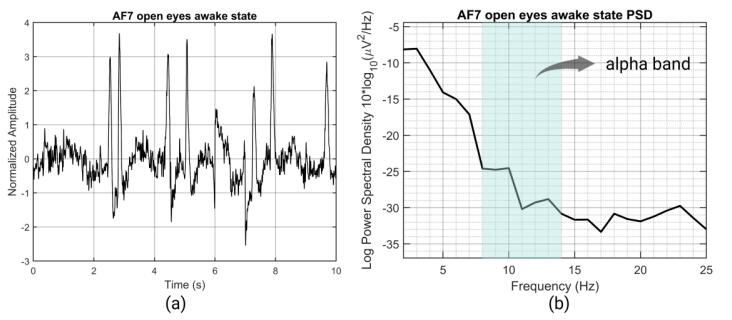
(a) waveform of the open eyes awake state (b) Power Spectral Density (PSD) of the open eyes awake state.

**Fig. 31:**
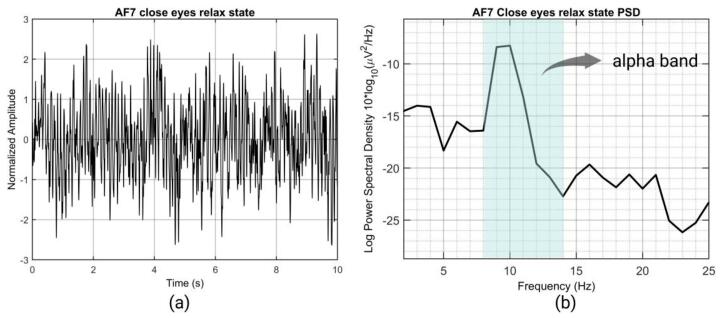
(a) waveform of the close eyes relax state (b) Power Spectral Density (PSD) of the close eyes relax state.

Two segments of EEG signals of 10 s each from the same subject at the AF7 site under open-eye and closed-eye conditions are presented. They are the 2–25 Hz energy spectrum of EEG under the open-eye awake state (Fig. 30), and the 2–25 Hz energy spectrum of EEG under the closed-eye relaxation state (Fig. 31). In the energy spectrum of the closed-eye relaxation state, the energy of the alpha rhythm (8–14 Hz) increases. The power spectral density calculation was provided by EEGLAB [36].

**Fig. 32:**
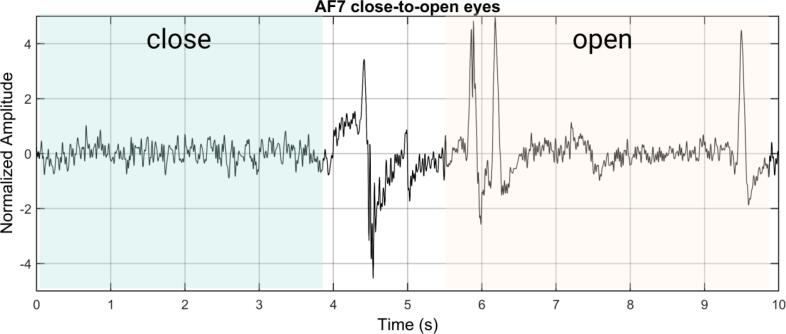
EEG waveforms of the subject transitioning from closed-eye relaxation to open-eye awake.

The subject was asked to alternate between the closed-eye relaxation state and the open-eye awake state, and EEG data from 0 to 10 s were captured (Fig. 32). The subject was in a closed-eye state for the first 4 s, and in an open-eye state from 6 to 10 s. It can be observed that during the closed-eye relaxation state, the EEG signals exhibit a regular waveform. The amplitude exhibits a regular change from small to large, and then from large to small, forming the so-called “shuttle” shape. This pattern is a typical feature of the alpha rhythm, which is prominent during relaxed states [37]. The duration of each “shuttle” is reported to be 1–2 s [37]. Upon opening the eyes (around the 10th second), it is mentioned that eye-blinking artifacts caused by the subject's blinking can be clearly observed in the time domain.

**Fig. 33:**
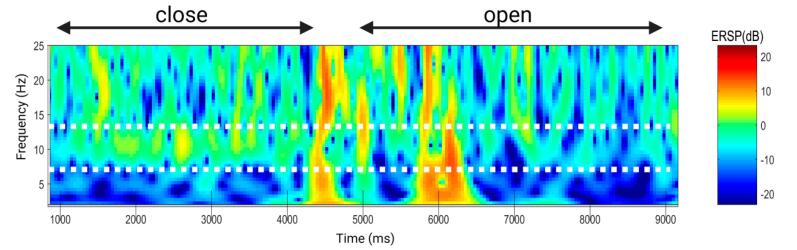
Time-frequency energy spectrum.

In the time–frequency energy spectrum (Fig. 33), it can be seen that the energy of the alpha rhythm in the closed-eye relaxation state has increased compared to the open-eye awake state.

### Characterization

Table 4

**Table 4:** Characterization summary.

Parameter	Characterization
Number of channels	4
Sampling rate	250 Hz
Sampling resolution	16bit
Gain	Adjustable from 360 to 2720
Input-referred noise	<0.9480 µVrms
Common Mode Rejection Ratio (CMRR)	> 96 dB
Input Impedance	No bias network: ∼5 GΩ
Bandwidth	0.5 Hz −45 Hz
Communication mode	BLE
Dimensions	45 mm x 30 mm x 12 mm
Weight	34 g (without battery)
Battery life	>4 h @ 250mAh 3.7 V lithium battery
VR Platform	Oculus Quest 2 or other VR platforms

## Ethics statements

Every subject has signed an informed consent before the experiment. The informed consent includes an introduction to the experiment, potential experimental risks, researcher's obligations and rights, subject's obligations and rights, compensation for participating in the experiment, and procedures for handling research-related injuries. Subjects have the right to withdraw from the experiment at any stage without any obligation.

## CRediT authorship contribution statement

**Zhiyuan Yu:** Conceptualization, Methodology, Data curation, Writing – original draft. **Shengwen Guo:** Writing – review & editing, Project administration, Supervision.

## Declaration of competing interest

The authors declare that they have no known competing financial interests or personal relationships that could have appeared to influence the work reported in this paper.
